# Acrosome reaction and chromatin integrity as additional parameters of semen analysis to predict fertilization and blastocyst rates

**DOI:** 10.1186/s12958-018-0408-0

**Published:** 2018-10-19

**Authors:** Pamela Tello-Mora, Leticia Hernández-Cadena, Jeimy Pedraza, Esther López-Bayghen, Betzabet Quintanilla-Vega

**Affiliations:** 1 Departamento de Toxicología, Centro de Investigación y de Estudios Avanzados del IPN, Av. IPN 2508, 07360 Ciudad de México, México; 2 Instituto Nacional de Salud Pública, Avenida Universidad 655, Santa María Ahuacatitlán, 62100 Cuernavaca, Morelos México; 3 Laboratorio de Investigación y Diagnóstico Molecular, Instituto de Infertilidad y Genética, Ingenes México, Carretera México-Toluca No. 5420, 05320 Ciudad de México, México

**Keywords:** Acrosome reaction, DNA fragmentation, Semen quality, Andrology diagnosis, In vitro fertilization

## Abstract

**Background:**

Traditional semen parameters have shown little to none predictive value for fertilization and blastocyst viability for a successful pregnancy. Therefore, the purpose of this study was to explore the usefulness of incorporating the acrosome reaction (AR) and chromatin integrity to conventional semen analysis to individually predict the fertile potential of sperm samples.

**Methods:**

A cross-sectional study was conducted in 69 participants undergoing IVF using oocyte donation. Semen samples were collected and evaluated for: AR [spontaneous (sAR) and induced (iAR)] by flow cytometry using anti-CD46-FITC, Acrosome Response to an Ionophore Challenge (ARIC), chromatin integrity by Sperm Chromatin Structure Assay (DNA Fragmentation Index-%DFI and High DNA Stainability-%HDS), WHO semen analysis, fertilization and blastocyst rates.

**Results:**

The participant age was 40.0 ± 6.1 years (66% were normozoospermic). Sperm morphology, sAR, iAR, and ARIC were associated with the fertilization (β = 3.56, R^2^ = 0.054; β = − 5.92, R^2^ = 0.276; β = 1.83, R^2^ = 0.150; and β = 2.10, R^2^ = 0.270, respectively, *p* < 0.05). A logit model was developed to calculate the probability of fertilization (≥ 60%) for each participant, using the sperm morphology and ARIC as independent variables, followed by ROC analysis to determine a cutoff probability of 0.65 (specificity = 80.6%, sensitivity = 63.2%). %DFI was inversely associated with the viable blastocyst rate (β = − 1.77, R^2^ = 0.057, *p* = 0.003), by the logit model and ROC analysis, a cutoff probability of 0.70 (specificity = 80.6%, sensitivity = 72.3%) was obtained to predict blastocyst viability (≥ 40%). There was no difference in the results with normozoospermic samples (*n* = 46).

**Conclusions:**

The incorporation of ARIC and %DFI allowed to obtain predictive models for high fertilization and blastocyst rates in an individualized way, being promising tools to improve the diagnosis of male fertility potential for research or assisted reproduction, even in men with unknown infertility.

## Background

The latest Center for Disease Control and Prevention (CDC) report indicates that about 11% of couples in reproductive age have fertility problems; global rates of male infertility are between 2.5–12% [1, 2]. Assisted reproduction techniques (ART) are responsible for 1.7–4% of pregnancies in developed countries, and in vitro fertilization (IVF) is the primary method that involves the incubation of oocytes with spermatozoa and the subsequent transfer of viable embryos in a woman’s uterus to achieve a pregnancy [1]. However, in cases of unexplained infertility or poor semen quality, low oocyte retrieval, or previous failed IVF cycles, highly complex techniques as the injection of one spermatozoon into the oocyte cytoplasm (ICSI) are performed [3]. Recently, new methods are available to improve the quality of the selected spermatozoon, such as hyaluronic acid (HA)-binding sperm selection (PICSI), magnetic-activated cell sorting (MACS) of non-apoptotic spermatozoa, and motile sperm organelle morphology examination (MSOME) techniques [4–6]. PICSI has shown some advantages over IVF and ICSI, such as higher percentage of viable and acrosome reacted cells and higher blastocyst rate in a porcine model [4]; and in humans, higher fertilization, blastocyst cleavage, and clinical pregnancy rates have been reported using the PICSI method [7]; however, some of these techniques increase the time of the fertilization procedure, have high costs or fail to determine genetic quality.

Due that semen quality assessed by WHO criteria does not fully predict male fertility [8], new methodologies have been developed to determine sperm quality and function, including proteomic analysis [9, 10], apoptotic markers [11], oxidative status [12, 13], DNA adducts [14], and DNA methylation status [15], obtaining good associations with male fertility. Unfortunately, these techniques are expensive and difficult to include in the operational process in andrology laboratories, and therefore have not had continuity.

A possible alternative to examine the sperm function is evaluating the acrosome reaction (AR), which is an essential event for sperm fertilization. The acrosome is considered a large secretory granule that contains several enzymes including glycohydrolases, proteases, esterases, acid phosphatases, and aryl sulfatases. The release of these hydrolytic enzymes degrade the zona pellucida allowing the spermatozoa to penetrate it and join with the oocyte in a process called AR, which is dependent on calcium (Ca^2+^); in human spermatozoa, the acrosome occupies 40 to 70% of the head [16]. The AR can be induced in vitro using a calcium ionophore (i.e. A23187), progesterone, phorbol myristate esters, and follicular fluid [17–22]. Specifically, the calcium ionophore transports extracellular Ca^2+^ into the cells or releases the ion from intracellular storages [23]. Ca^2+^ plays a key role in the activation of signaling pathways of the sperm capacitation and posterior AR [24], for example, the entry of Ca^2+^ induces the inactivation of the PI3 kinase, resulting in the activation of serine/threonine protein kinases and the sperm exocytosis [25]. After the induction of the AR, the presence/loss of acrosome can be detected by the presence of proteins in the acrosome region, as the CD46 protein, which is exposed in the internal acrosome membrane once the proteolytic content is released [26]. It has been reported that the in vitro AR evaluation might predict 30 to 50% of fertilization in patients under IVF [27–29]. On the other hand, it has been reported that some environmental-related factors, such as diet, physical activity, and exposure to environmental contaminants like pesticides and metals alter the AR and therefore the sperm function [30–32].

Normal sperm chromatin is a requisite for the expression of full male fertility potential because sperm chromatin abnormalities (abnormal packing, chromatin integrity, or DNA fragmentation) are common among infertile men [33]. One of the most promising techniques associated with male fertility potential is the Sperm Chromatin Structure Assay (SCSA), which measures the susceptibility of in situ acid-induced sperm DNA denaturation by staining with the fluorescent dye acridine orange. The red/green fluorescence allows to evaluate the integrity of the sperm DNA (%DFI, DNA Fragmentation Index) and the chromatin structure (%HDS, High DNA Stainability) by flow cytometry [34, 35]. SCSA parameters have shown important implications in pregnancy lost and embryonic viability [36, 37], and do not correlate with WHO semen parameters, especially in normozoospermic men with unexplained infertility [38]. The etiology of DNA damage is multifactorial and can be related to diet, exposure to xenobiotics, such as pesticides, and psychological stress (anxiety), among others that are considered poor semen quality risk factors and are negatively associated with male fertility [13, 31, 39, 40]. Even though the integrity of DNA is involved in male fertility and it does not always correlate with WHO semen parameters, only a few andrology laboratories include SCSA or any other DNA integrity assay in their routine analyses [41].

To offer a better prediction of pregnancy success in andrology laboratories and in search of risk factors of poor semen quality, the objective of this study was to develop individual predictive equations of the fertilization and blastocyst rates, incorporating additional parameters such as the AR and DNA fragmentation as the sperm function/quality variables.

## Methods

### Participants

A transversal epidemiological study was conducted in men attending a fertility clinic, the Ingenes Institute in Mexico City. Two criteria were considered to include the participants: men < 55 years old undergoing an IVF treatment and with the indication of oocyte donation to avoid the female factor, and we excluded the azoospermic patients, or those whose semen sample was not enough to provide an aliquot without compromising the IVF treatment. Oocyte donors were women under 30 years, healthy and with proven fertility (own pregnancy or by donation in previous cycles). This study was approved by the Ethics Committee of the Ingenes Institute, and written informed consent was obtained from all participants. A total of 170 men were invited, and 153 accepted to participate (response rate = 90%); however, 84 participants were not included because their semen samples were not enough to provide an aliquot for the analyses or their IVF cycles were canceled; therefore the final samples came from 69 participants.

### Semen sample

Semen samples were collected by masturbation (after 3–5 days of abstinence) on the day of the IVF procedure. After liquefaction, samples were analyzed according to WHO criteria for volume, time of liquefaction, pH, leukocyte concentration, sperm concentration, progressive motility, morphology (by the Kruger criteria), and viability [42]. Immediately after the semen analysis was completed, one aliquot of semen was taken and 10 × 10^6^ spermatozoa were used for the AR analyses (in fresh) and 2 × 10^6^ spermatozoa were frozen (− 70 °C) for the SCSA analysis.

For sperm capacitation, spermatozoa were subjected to Percoll gradient (45–90%) prepared using PureSperm 100 (Nidacon International, Mölndal, Sweden) and human tubal fluid medium (HTF, Irvine Scientific, Santa Ana, CA, USA). The sample was washed with 1 ml HTF medium supplemented with 10% human serum albumin (HSA, Invitrocare, Frederick, MD, USA), and centrifuged at 1500 rpm/10 min. The supernatant was removed, and 400 μl of HTF-10% HSA medium was added and incubated for 2 h/room temperature (RT).

### Ovarian stimulation for oocytes donation

Female donors were subjected to ovarian stimulation consisting in a daily administration (8–14 days; average = 10 days) of gonadotropin-releasing hormone (GnRH) antagonists (as available): 0.25 mg/day Cetrorelix (Cetrotide, Merck Laboratory Darmstadt, Germany) or 0.25 mg/day Ganirelix acetate (Orgalutran Laboratory MSD, Kenilworth, NJ, USA) in the luteal phase after menses. Gonadotropins were adjusted according to serum estradiol levels and vaginal ultrasound measurement of follicular diameter every 2–3 days. Stimulation was prolonged until the diameter of leading follicles was > 18 mm (18–22 mm). Then, recombinant human chorionic gonadotropin (hCG) (Choragon 1000 IU, Laboratorio Ferring, Saint-Prex, Switzerland) was administered and oocytes were retrieved after 36 h with ultrasound guidance. All 14–18 mm follicles were aspirated, and an average of 12.34 ± 4.85 oocytes were assigned to each participant.

### Embryo culture

Semen samples were prepared by density gradient centrifugation, and fertilization was performed as follows: the donated oocytes (12.3 ± 4.8 per participant) were inseminated 4 h after harvesting with approximately 100,000 capacitated spermatozoa per oocyte in a plate with Global Total medium (LifeGlobal; St Petersburg, FL, USA) and mineral oil and were incubated for 19 h in 8% CO_2_, 5% O_2_ at 37 °C; fertilization was considered as positive by the formation of pronuclei. Fertilization was considered as normal if two pronuclei and two polar bodies were identified, while oocytes without visible pronuclei were unfertilized, and those with a single pronucleus or more than two pronuclei were classified as abnormally fertilized.

Embryos were cultured in Global Total medium (LifeGlobal, St Petersburg, FL, USA) and incubated at 37 °C in 8% CO_2_. Embryo’s quality was monitored according to morphology criteria: number of blastomeres and division rhythm, percentage and type of fragmentation, visualization of nuclei, presence of cytoplasmic halo or vacuoles in the cytoplasm, zona pellucida grossor, and degree of compaction and symmetry: blastomere size as a function of the stage. The embryos graded 1 and 2 were considered with optimal quality and maximum/good implantation capacity, embryos of grade 3 had intermediate quality, and embryos graded 4 were considered of poor quality with a low probability of implantation. In this study, embryos of grades 1 to 3 were considered viable and suitable for transfer. An embryologist monitored and recorded the information about fertilization and embryo quality daily.

### Acrosome reaction evaluation

To evaluate the AR, the CD46 protein that is exposed in the internal acrosome membrane once the reaction occurs [26] was detected using anti-CD46-FITC. An aliquot of semen (10 × 10^6^) was capacitated as previously described, and the spermatozoa were separated into 4 tubes (100 μl each) to evaluate the spontaneous AR (sRA) and the induced AR (iAR) in duplicate.

To evaluate the sAR (representing the premature loss of the acrosome), 10 μl of anti-CD46-FITC antibody (BD Pharmingen, San Jose, CA, USA) was added to the cells and incubated for 30 min/RT in the dark, then the cells were washed with HTF medium and centrifuged at 1500 rpm/5 min. The pellet was suspended in 300 μl of PBS and propidium iodide was added as a viability marker (1 μg/ml, Sigma-Aldrich, San Luis, MO, USA). For the iAR evaluation (representing the acrosome function in response to an inducer), spermatozoa were washed with 500 μl of HTF without HSA (which has affinity for the inducer) [43]. Once the supernatant was removed, a calcium ionophore (A23187, stock of 1 mg/ml in DMSO; Sigma-Aldrich, San Luis, MO, USA) was added to a final concentration of 10 μM/1 h/RT; then, cells were washed with HTF and incubated with anti CD46-FITC and propidium iodide as described above. Flow cytometry was performed with the BD Accuri™ C6 cytometer, and the fluorescence of the anti-CD46-FITC antibody (green, 488 nm excitation) and the propidium iodide (red, excitation at 575 nm) was recorded. One thousand events were evaluated in duplicate. Unstained cells from each participant were analyzed to establish the negative sperm population. Spermatozoa without acrosome were represented by cells positive to FITC (acrosome absent) and negative to propidium iodide (alive) and were analyzed with the software BD Accuri C6. The ARIC (Acrosome Response to a lonophore Challenge) score was calculated by subtracting the sAR from the iAR (ARIC = iAR-sAR).

### Sperm chromatin structure assay (SCSA)

The SCSA was performed according to Evenson [44]. Spermatozoa aliquots (2 × 10^6^ spermatozoa) were thawed in a 37 °C water bath and suspended in 1 ml of 1X TNE buffer (10X: 0.01 M Tris-HCl, 0.15 M NaCl, 1 mM EDTA, pH 7.4); 200 μl of each sample were added to 400 μl of a detergent solution (0.1% Triton X-100, 0.15 M NaCl, 0.08 M HCl, pH 1.2) for 30 s, and 1.2 ml of acridine orange staining buffer (6 μg/ml acridine orange, 37 mM citric acid, 126 mM Na_2_HPO_4_, 1 mM disodium EDTA, 0.15 M NaCl, pH 6.0) was immediately added. After 3 min of incubation, cells were analyzed by flow cytometry in a BD FACSCalibur™; the fluorescence intensity of 5000 events (excitation at 488 nm) was recorded for double-stranded (green: native) and single-stranded (red: damage) DNA. Results were analyzed using the SCSA® software. The DNA damage was represented by the %DFI parameter, and the sperm with abnormal chromatin condensation by the high DNA stainability (%HDS). As quality control, a sample with known DFI and HDS was evaluated each day of analyses.

### Statistical analyses

Results were expressed as the means ± SD. Lineal regression analysis was performed to determine the association between the AR, SCSA or WHO parameters and the IVF outcomes (fertilization and blastocyst viability). We obtained two logit models with the variables of IVF and as predictor variables those associated in the lineal regression (used as continuous variables). Once the logit model was obtained, these values were used in the logistic function to develop the predictive equation and calculate the probability of fertilization ≥60% and viable blastocysts ≥40%:

$$ \widehat{p}=\frac{e^{\beta_0+{\beta}_1{X}_1+{\beta}_2{X}_2\dots {\beta}_n{X}_n}}{1+{e}^{\beta_0+{\beta}_1{X}_1+{\beta}_2{X}_2\dots {\beta}_n{X}_n}} $$

Where, β_0_ represents the basal probability in case X_1_, X_2…_ X_n_ have zero values; β_1_, β_2…_ β_n_ are the coefficients of the predictive variables in the model; while X_1_, X_2…_ X_n_ are the results obtained of each participant.

The Receiver Operating Characteristic (ROC) curve was used to determine which cutoff would provide the best trade-off between sensitivity and specificity, in this case they were 0.65 for fertilization and 0.70 for blastocyst prediction. The area under the ROC curve (AUC) was calculated using the method described by Hanley and McNeil [45]. To determine the dichotomization of the logit model, we performed several models with different cutoffs in the IVF variables (at 5% intervals), as well as in the ROC analysis (0.5 basal and then increments at intervals of 0.05), at the end, the model with the best specificity and sensitivity was obtained. The final dichotomization of fertilization at ≥60% and blastocysts with optimal morphology at ≥40% was based on the average values observed in this study and that is considered as a successful result in the clinic. Once the models were obtained, we replaced the results of each participant (ARIC, morphology, and %DFI) in the predictive equation and the percentage of participants whose IVF result matched with the prediction was calculated, this was considered as the accuracy of the test. The STATA V.12.0 (Stata Corp, College Station, TX, USA) software was used for all analyses. A level of *p* ≤ 0.05 was considered significant.

## Results

### General characteristics of participants

The general characteristics of the participants are shown in Table 1. The mean age of participants was 40.0 ± 6.1 years, Bachelor’s was the main academic degree (47.8%), and 56.5% were residents of the metropolitan area of Mexico City; 11.6% had a BMI in the normal range, 49.3% regularly exercised, and 56.5% frequently consumed antioxidants. A minimum percentage of participants were smokers (5.8%), and few had a diagnosis of a disease (4.3% hypertension, 8.7% varicocele, and 7.2% a genital infection).

**Table 1:** Sociodemographic and health characteristics of the participants

Variable	% (n)
Age (years; mean + SD)	40.0 ± 6.1(69)
Education	
Elementary or middle school	7.2 (5)
High school	14.5 (10)
Bachelor’s degree	47.8 (33)
Post-graduate	30.4 (21)
Residence in the metropolitan area of Mexico City (%)	56.5 (39)
Body mass index (BMI)	
Normal (≤ 24.9)	11.6 (8)
Overweight (≥ 25)	52.2 (36)
Obesity (≥ 30)	36.2 (25)
Current smokers	5.8 (4)
Consumption of antioxidants^a^	56.5 (39)
Regular exercise^b^	49.3 (34)
Hypertension^c^	4.3 (3)
Genital infections^c^	7.2 (5)
Varicocele^d^	8.7 (6)

^a^At least 3 times per week (as vitamins E, A, selenium, etc.). ^b^At least 1 h per day

^c^Diagnosis in the last 3 months. ^d^Current diagnosis. *n* = 69

### Sperm function, quality parameters, fertilization and viable blastocyst

Table 2 shows the results of semen parameters and IVF outcomes. For semen quality parameters, 66.7% of participants were normozoospermic, 26.1% teratozoospermic, 5.8% asthenoteratozoospermic, while only 1.4% were hypozoospermic according to WHO criteria. The percentage of sAR (premature loss of acrosome) had a mean value of 3.16 ± 1.78%, and the iAR (functional acrosome) of 13.42 ± 4.25%, while the mean value of ARIC was 10.26 ± 4.98%. Data of the integrity of sperm chromatin showed a mean value of %DFI of 21.79 ± 6.29 and 7.11 ± 3.55 of %HDS. Regarding the DNA fragmentation, 33.3% of participants had values of %DFI above that considered as of poor fertility in the literature (≥ 25%) and the blastocysts morphologically optimal of this group was < 42%; in relation to %HDS, none of the participants had values above 25%, which is established as the threshold for pregnancy success [44]. Results of the IVF cycle showed that the number of donated oocytes was 12.3 ± 4.8 per participant, and the rate of fertilized oocytes per participant was 60.3 ± 20.2%, from which 49.8 ± 31.3% developed a viable blastocyst (grades 1 to 3) to transfer. The pregnancy result was not considered in this study, since the differences in the receptor women couldn’t be taken into consideration and it was out of the scope of this study.

**Table 2:** Sperm quality and function parameters, and IVF outcomes of the participants

Variable	Mean ± SD	Range
WHO parameters		
Volume (ml)	2.8 ± 1.2	1.2–6.6
Concentration (× 10^6^/ml)	77.8 ± 44. 2	16–230
Total count (× 10^6^)	231.8 ± 177.9	36–1016
Progressive motility (%)	52.6 ± 16.3	6–78
Normal morphology (%)	3.7 ± 1.5	1–7
pH	8.4 ± 0.57	7–9
Viability (%)	75.6 ± 10.7	53.8–93.4
Leukocytes (× 10^6^/ml)	0.5 ± 0.61	0–3.5
Sperm quality and function parameters (%)		
sAR	3.2 ± 1.8	0.4–9.02
iAR	13.4 ± 4.2	4.7–26.9
ARIC	10.3 ± 4.9	0.8–24
DFI	21.8 ± 6.3	8–37
HDS	7.1 ± 3.5	3–16
IVF cycle outcome		
Donated oocytes/participant	12.3 ± 4.8	2–24
Fertilized oocytes (%)^a^	60.3 ± 20.2	18.7–100
Optimal blastocysts (%)^b^	49.8 ± 31.3	0–100

sAR = Spontaneous acrosome reaction, iAR = Induced acrosome reaction, ARIC = Acrosome response to an ionophore challenge. DFI DNA fragmentation index, HDS = High DNA stainability. ^a^Defined as oocytes showing two pronuclei and two polar bodies. ^b^Compared to fertilized oocytes. *n* = 69

### Bivariate associations of semen parameters with fertilization and blastocyst rates

The linear regression analysis (Table 3) revealed a positive association between the percentage of normal morphology and the fertilization rate (β = 3.56, R^2^ = 0.054, *p* < 0.05); no other WHO semen parameter was associated with the fertilization rate. Significant associations were observed with the three AR parameters; a negative association was obtained with sAR, a decreased of 5.92% in the fertilization rate was observed for each increase in sAR (β = − 5.92, R^2^ = 0.276, *p* = 0.0001), while positive associations were observed with iAR (β = 1.83, R^2^ = 0.150, *p* = 0.0001) and ARIC (β = 2.10, R^2^ = 0.270, *p* = 0.0001). ARIC represents the functional spermatozoa; therefore, subsequent analyses were performed only with the ARIC. No associations were observed between SCSA parameters and the fertilization rate.

**Table 3:** Bivariate associations between sperm parameters and IVF outcomes

Variable	Beta coefficient /R^2^	
	Fertilization (%) (CI 95% β)^a^	Viable embryos (%) (CI 95% β)^a^
WHO parameters		
Volume (ml)	0.86 / 0.003 (− 3.09, 4.81)	−2.55 / 0.010 (− 8.67, 3.56)
Concentration (× 10^6^/ml)	− 0.02 / 0.000 (− 0.10, 0.11)	0.09 / 0.020 (− 0.07, 0.26)
Total count (× 10^6^)	0.00 / 0.003 (− 0.02, 0.03)	− 0.04 / 0.000 (− 0.03, 0.04)
Motility (%)	3.19 / 0.000 (− 0.31, 0.37)	− 0.13 / 0.003 (− 0.66, 0.40)
Morphology (%)	**3.56 / 0.054** **(0.05, 7.18)** ^*^	−2.75 / 0.013 (− 8.48, 2.98)
Viability (%)	0.20 / 0.012 (− 0.24, 0.66)	− 0.20 / 0.004 (− 0.90, 0.50)
pH	− 0.92 / 0.000 (− 9.29,7.44)	−5.79 / 0.011 (− 18.71, 7.12)
Leukocytes (× 10^6^/ml)	− 3.93 / 0.015 (− 11.66, 3.80)	−9.35 / 0.035 (− 21.23, 2.52)
Sperm quality and function parameters *(%)*		
sAR	**−5.92 / 0.276** **(− 8.26, − 3.58)** ^***^	**− 4.19 / 0.057** **(− 8.33, − 0.04)** ^*^
iAR	**1.83 / 0.150** **(0.76, 2.89)** ^***^	−0.87 / 0.014 (− 0.90, 2.65)
ARIC	**2.10 / 0.270** **(1.26, 2.94)** ^***^	−0.18 / 0.035 (− 0.32, 2.68)
DFI	0.18 / 0.003 (− 0.59, 0.96)	**−1.77 / 0.127** **(− 2.91, − 0.64)** ^**^
HDS	−0.65 / 0.013 (− 2.02, 0.72)	−0.52 / 0.003 (− 2.66, 1.61)

sAR = Spontaneous acrosome reaction, iAR = Induced acrosome reaction, ARIC = Acrosome response to an ionophore challenge, DFI = DNA fragmentation index, HDS = High DNA Stainability. ^a^CI= 95% confidence interval. In bold, the values with statistical significance are highlighted. *p = 0.05, **p = 0.003, ***p < 0.0001. n = 69

For the blastocyst rate, there was no association with the WHO semen parameters. The sAR and %DFI were negatively associated with the blastocyst rate (β = − 4.19, R^2^ = 0.057, *p* = 0.05 and β = − 1.77, R^2^ = 0.127, *p* = 0.003, respectively). The %HDS was not associated with blastocyst formation.

### Prediction of success: Fertilization and blastocyst rates

The fertilization rate was dichotomized at 60% for the logit model, using the predictive variables that showed an association in the lineal regression analysis (ARIC and morphology) (Table 4). No other confounding variable was significant in the model (including age, BMI, etc.). From the β coefficients obtained in the logit model, a predictive equation (see Statistical analyses) was developed for a probability of having a fertilization rate ≥ 60%. Where, β_0_ = − 4.58 (corresponding to the constant value of the model), β_1_ = 0.24 (corresponding to the coefficient of the variable ARIC), and β_2_ = 0.59 (corresponding to the coefficient of the variable morphology); while X_1_ = ARIC and X_2_ = morphology, were taken from the results obtained from each participant.

**Table 4:** Logit model for a successful fertilization

Fertilization ≥60%	Beta coefficient (CI)^a^	SE	*p* value
% ARIC	0.24 (0.10–0.37)	0.06	0.000
% Morphology	0.59 (0.14–1.04)	0.23	0.01
Constant	−4.58 (−7.17 to −1.98)	1.32	0.001

ARIC = Acrosome response to an ionophore challenge, ^a^95% confidence interval. ROC cutoff = 0.65. AUC = 803. Sensitivity = 63.2%. Specificity = 80.6%. Positive predictive value = 80%. Negative predictive value = 64.1%. *n* = 69. Constant = βo from the logit model (Ln(basal probability of Fertilization ≥60% if ARIC and % morphology are equal to zero))

When the probabilities were plotted in a ROC curve (AUC = 0.803, 95% CI: 0.717 to 0.888, *p* < 0.001), the best cutoff obtained was 0.65, with a specificity = 80.6% and sensitivity = 63.2%, and the positive predictive value was 80% and the negative predictive value was 64%; using this cutoff value, the fertilization result in each participant was re-evaluated in our database resulting in a validated test accuracy of 74%.

The blastocyst rate was dichotomized at 40% for the logit model using %DFI as a continuous variable (Table 5), and the predictive equation (see Statistical analyses) was developed. Where, β_0_ = 5.11 (corresponding to the constant value of the model), β_1_ = − 0.19 (corresponding to the coefficient of the variable %DFI), and X_1_ = %DFI taken from the results obtained from the SCSA. The association observed in the linear regression analysis between sAR and viable blastocyst lost the significance in the logit model.

**Table 5:** Logit model for success of having a viable blastocyst

Viable embryos ≥40%	Beta coefficient (CI)^a^	SE	*p* value
%DFI	−0.19 (− 0.37 to − 0.08)	0.05	0.001
Constant	5.11 (2.09–8.51)	1.36	0.000

DFI = DNA fragmentation index. ^a^CI: 95% confidence interval. ROC cutoff = 0.70. AUC = 0.773. Specificity = 68.2%. Sensitivity = 72.3%. Positive predictive value = 82.9%. Negative predictive value = 53.6%. Constant = βo from the logit model (Ln(basal probability of viable embryos ≥40% if %DFI is equal to zero)) n = 69

When the probabilities were plotted in a ROC curve (AUC = 0.773, 95% CI: 0.673 to 0.873, *p* < 0.0002), the best cutoff obtained was 0.70 with a specificity = 68.2% and sensitivity = 72.3%, and the positive predictive value was 83% and the negative predictive value was 54%. Using this cutoff value, the test accuracy considering our database was of 72%.

Additionally, the association between ARIC and %DFI and the IVF outcomes was explored in those participants considered as normozoospermic (*n* = 46), according to the WHO criteria. The linear regression analyses showed a negative association between the fertilization rate and sAR (β = − 7.39, 95% CI: -10.34 to − 4.44, *p* = 0.0001), and positive associations with iAR (β = 2.30, 95% CI: 1.15 to 3.45, *p* = 0.0001) and ARIC (β = 2.57, 95% CI: 1.67 to 3.47, *p* = 0.0001); while a decrease in the blastocyst rate was observed when %DFI increased (β = − 2.18, 95% CI: -3.40 to − 0.95, *p* = 0.001). These results agree with those obtained with all participants.

## Discussion

The aim of this study was to evaluate if sperm quality and function parameters such as AR and chromatin integrity were useful to develop an equation to individually predict successful fertilization and blastocyst rates. We showed that ARIC correlated and predicted the fertilization rate, while %DFI correlated and predicted the blastocyst rate. Due to the low time and cost to perform these sperm evaluations their incorporation into the conventional analysis is feasible.

Results obtained in the present study showed that sperm morphology and AR data showed a positive association with the fertilization rate; sperm morphology has been already considered a good predictor of fertility, without a female infertility factor [46–48]. Other semen parameters have shown little to none predictive value [8, 49, 50]. In previous studies, the AR assessed in human semen samples by different techniques showed a good correlation with fertilization [51–53]; however, there was not continuity to this finding. Using the CD46 antibody and the calcium ionophore A23187 as inducer to evaluate the acrosome status, the iAR values obtained in this study (13.4 ± 4.2) are similar to those obtained in a study performed in healthy donors using the same technique (11.6 ± 2.1%) [21], and are lower than those detected by FITC-PSA lectin in subfertile men (19.0 ± 11.80%) [17]. The sAR, iAR, and ARIC parameters were highly associated with the fertilization rate. In this study, we chose the ARIC for the predictive equation because it represents the response of spermatozoa to a stimulus and their ability to undergo the AR.

The AR is an event mediated by complex signaling pathways that include intracellular Ca^2+^ and other ions, sperm plasma membrane fluidity associated with cholesterol content, and the formation of SNARE (Soluble N-ethylmaleimide-sensitive attachment protein receptors) protein complexes, among others, that cause the fusion and coupling of the plasma membrane with the external acrosome membrane [24, 54]. After the AR occurs, some proteins in the membrane of the equatorial and post-acrosomal regions are modified, such as IZUMO, CRISP, SPESP1, Fertilin, etc., which are necessary for the posterior fusion with the oocyte [55]. These modifications cannot be present in the spontaneous-reacted spermatozoa, because it is considered as a false AR produced by complex mechanisms, including the time from ejaculation to the IVF procedure, even by long processing in IVF. In this way, the AR can be modified by several factors, internal and external, through different molecular mechanisms. For instance, it is known that lead causes a premature AR [32] or a reduced induction of the AR [56]; this metal enters to sperm cells through Ca^2+^ channels altering its homeostasis, and causes DNA damage and oxidative stress altering multiple events of the AR, including a downregulation of the acrosin activity, decreased intracellular Ca^2+^ concentration, and elevated calmodulin concentration [56]. Obesity is another factor that impairs the AR due to altered circulating levels of estradiol and low response to progesterone, both being inducers of the AR [57]. In most cases, including obesity and metal exposures, the determinant factor for a defective acrosomal function is oxidative stress, which results in multiple molecular alterations, such as protein carbonylation and lipoperoxidation, thereby in sperm damage to cellular membranes or proteins involved in pathways regulating acrosomal exocytosis, such as protein kinase B (Akt), serine/threonine kinases, G proteins, phospholipase C, CatSper channels, etc. [58, 59]. ARIC was reported as a sperm function parameter in the early 90’s [17, 28], but has not been included in andrology laboratories as a routine test. The utility of the AR to predict fertilization is variable in the literature, and in the most recent reports, the success of prediction was low and depended on the study design and type of detection (by using AR specific fluorescence probes vs. sperm morphology). Söderlund & Lundin (2001) reported that 7% of acrosome index could predict 50% of fertilización in 81 patients undergoing an IVF (specificity = 62%, sensibility = 81%); however, they only included patients with a poor prognosis and the acrosome evaluation was made with the Papanicolau morphology test [60]. On the other hand, more recently, Wiser et al. found that < 10% of sAR predicted > 35% fertilization in preliminary results in 40 patients (specificity 50% and sensitivity 94%), using a fluorescein-conjugated *Pisum sativum* agglutinin to detect the acrosome [29]. Here, the combination of ARIC and sperm morphology allowed us to obtain an individual prediction for at least 60% of fertilization with good specificity (80.6%) and sensitivity (63.2%). When these two parameters were applied to our equation with the participant results, 74% of cases were predicted as positive, i.e., the predictive data agreed with the IVF outcomes obtained. Therefore, by applying the predictive equation proposed in this study, using the ARIC and morphology, if an individual’s semen sample has a prediction value of ≤0.65 (probability cutoff), that is equivalent to predict a fertilization under 60%, and an ART procedure could be suggested by the andrologist, including the donation of male gametes, or can contribute to evaluate the role of external factors on the fertilization capacity in a population.

Genetic damage is one of the causes of unexplained male infertility when conventional semen parameters are normal [61]. DNA fragmentation has been widely associated with natural and assisted reproduction success (IVF and ICSI) and is one of the latest most studied parameters of sperm quality; it has been associated with decreased embryo quality and viability [36, 62–65], implantation failure [66, 67], and pregnancy failure [37, 68–72]. Here, there was an inverse relationship between DNA damage (%DFI) and blastocyst rate. The SCSA is specifically validated for the evaluation of sperm chromatin and DNA integrity, with current reference values of %DFI ≥ 25 and %HDS ≥ 25 to diagnose poor sperm quality, and are mainly associated with a high time to natural pregnancy or intrauterine insemination failure [44]. In the present study, the highest %HDS value was 16% (*n* = 1), this is, none of the participants had an altered chromatin stability, while 33.33% of participants showed a high DNA fragmentation with %DFI values higher than 25 and the consequent blastocyst viability of this group was lower than 42%. Using a blastocyst rate of ≥40%, a value considered clinically acceptable in fertility clinics, we calculated the specificity (68.2%) and sensitivity (72.3%) of the predictive model using a probability cutoff of 0.70. Therefore, when the %DFI parameter is used in the equation and gives a value ≥0.70, the semen sample will potentially have a probability of ≥40% to produce an optimal blastocyst. In this study, no association was observed between %DFI and the fertilization rate, as expected, because the sperm’s genetic material is activated in the 4-cell phase. Thus a spermatozoon with high genetic damage can fertilize and form the pronucleus, but the damage may manifest before or during blastocyst formation. The oocyte is responsible of the post-fertilization repair; however, when this process is not successful, it results in arrested development and failed pregnancy [73–75], or it increases the number of mutations with consequences to the offspring, such as ﻿congenital malformations, ﻿childhood cancer, and neurological disorders [59]. DNA fragmentation of germ cells can occur as a result of apoptosis, by chromatin compaction during the replacement of histones by protamines [33], by oxidative stress mechanisms derived from lifestyle or exposure to xenobiotics like pesticides, among others, which are considered risk factors of poor semen quality [13, 31, 39]. Oxidative stress, mainly in the epididymis and during transport in the seminal fluid, is the main responsible for the DNA damage caused by the generation of oxidized bases as 8-hydroxy,2′ deoxyguanosine (8-OHdG), and the subsequent strand breaks due to the lack of repair mechanisms [14, 76]. In addition, exposure to radiation or toxic agents induces DNA damage, which stimulates the synthesis of p53 proteins that are cell sensors activating pro-apoptotic proteins to trigger apoptosis [77].

It is important to highlight that beyond the knowledge that %DFI is a good predictor of successful natural and assisted fertilization outcomes, its use in our equation will allow to individually predict the success of at least 40% of having morphologically optimal blastocysts, which can be crucial to suggest an ART to a couple; the advantage of using SCSA is that it can be performed in advance of any treatment without disturbing the sample for future procedures [44], allowing the decision of the best assisted reproduction treatment, or to suggest a treatment to improve the semen parameters. We are aware that the comparison of our data with alternative techniques that evaluate DNA fragmentation is necessary, since SCSA software is not available for free.

This study included participants with a variety of semen quality according to WHO, from 28 to 55 years old and with different sociodemographic characteristics and lifestyles (e.g. overweight, exercise, etc.); although they had similarities, participants also represented a heterogeneous population, which may favor the usefulness of our predictive equation in other men populations with fertility problems. Additionally, the associations obtained between the fertilization and blastocyst rates and the AR and %DFI were also observed in only normozoospermic participants, suggesting that these sperm function parameters are tools that can successfully predict male fertility even in participants with unexplained infertility. Although this study was performed in an IVF clinic to help with the subfertility problems of participants, our equations can be applied in any andrology laboratory to know the prediction of each patient of either his fertilization or blastocyst rates. This may allow them to consider the best procedure to have a child based on their own parameters.

Our study contains some limitations. The validation of the predictive equations was done with our study database, obtaining 74 and 72% coincidence with the fertilization rate and blastocyst viability results, respectively. It is necessary to perform the validation in other populations undergoing an IVF process. It would also be optimal to know the inter-assay variations of AR and %DFI of each participant, understanding that the factors affecting these parameters would allow a better prognosis; however, determining how these values vary was not in the scope of this study. Additionally, it was assumed that the oocytes were of optimal quality and had no alterations, since they were donated by healthy women with proven fertility. Finally, our study evaluated the sperm morphology, ARIC and %DFI, but this does not necessarily imply that other parameters of sperm function that may be developed in the future may be good predictors as well. Nevertheless, the low cost and time to obtain the results of the variables evaluated in this study favor the use of the proposed methods and predictive models.

## Conclusions

The incorporation of the AR evaluation, in addition to sperm morphology, allowed to predict a high fertilization rate (60%) individually, and the sperm DNA fragmentation (%DFI) predicted the blastocyst viability in men with fertility problems; both predictions will give an acceptable prognosis of a successful pregnancy in the lack of a female infertility factor. A highlight aspect of the predictive equations presented here is that they may help in the individual diagnosis and therefore in their decision to undertake an ART and be successful in having a baby, even in cases of unexplained male infertility, which would reduce unnecessary stress to couples, costs and time. Additionally, the use of this predictions can be useful to identify a potential risk of fertility problems during a conventional semen analysis or in research studies.

## Abbreviations

AR: Acrosome Reaction
ARIC: Acrosome Response to an Ionophore Challenge
ART: Assisted Reproductive Technique
AUC: Area Under the Curve
DFI: DNA Fragmentation Index
HDS: High DNA Stainability
HTF: Human Tubal Fluid Medium
iAR: Induced Acrosome Reaction
ICSI: Intra Cytoplasmic Sperm Injection
IVF: In Vitro Fertilization
ROC: Receiver Operating Characteristic Curve
RT: Room Temperature
sAR: Spontaneous Acrosome Reaction
SCSA: Sperm Chromatin Structure Assay
